# A re‐examination of the circumscription of *Saxifraga mengtzeana* (Saxifragaceae)

**DOI:** 10.1002/ece3.9886

**Published:** 2023-03-12

**Authors:** Xin‐Jian Zhang, Richard J. Gornall, Zhuo‐Xin Zhang, Jun‐Tong Chen, Hang Sun, Tao Deng

**Affiliations:** ^1^ CAS Key Laboratory for Plant Diversity and Biogeography of East Asia, Kunming Institute of Botany Chinese Academy of Sciences Kunming China; ^2^ University of Chinese Academy of Sciences Beijing China; ^3^ Department of Genetics & Genome Biology University of Leicester Leicester UK; ^4^ College of Forestry and Landscape Architecture South China Agricultural University Guangzhou China

**Keywords:** China, morphology, phylogeny, Saxifragaceae, taxonomy

## Abstract

In the *Flora of China* account of *Saxifraga mengtzeana* Engl. & Irmsch., eight synonyms were attributed to it and one variant, recognized as *Saxifraga epiphylla* Gornall & Ohba, was split from it. This study reevaluates the taxonomic status of some of the synonyms and of the segregated species in light of new evidence presented here. Morphological and molecular evidence demonstrate that collections from northwestern Yunnan and Sichuan are genetically differentiated from those in southeastern Yunnan and neighboring Guangxi. Observations in the field and in cultivation show that the peltate petiole attachment diagnostic of *S. mengtzeana* var. *peltifolia* Engl. & Irmsch. is developmentally labile. Similar observations combined with molecular data show that viviparous phenotypes, formerly treated as *S. epiphylla*, although largely under genetic control, occur sporadically throughout the ranges of both northern and southern taxa. Collections from northwestern Yunnan and Sichuan are best recognized as *Saxifraga geifolia* Balf.f., whereas those from southeastern Yunnan and neighboring Guangxi are *S. mengtzeana*. Peltate‐leaved variants of the latter are given no status and are relegated to complete synonymy. Viviparous phenotypes of *S. mengtzeana* and *S. geifolia* are recognized at the rank of forma.

## Graphical abstract

Integrating morphological, geographical, and molecular evidence, we reinterpret the taxonomic and phylogenetic status of *Saxifraga mengtzeana* and related taxa. Our data suggest that Saxifraga epiphylla is part of a polymorphism and should be synonymized under *Saxifraga mengtzeana* and that *Saxifraga geifolia* should be distinct rather than conspecific with *S. mengtzeana*.

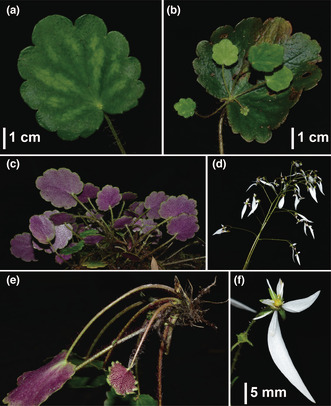

## 1 INTRODUCTION

*Saxifraga* L., the largest genus in Saxifragaceae, comprises more than 440 species that are widely distributed throughout arctic and montane regions of the Northern Hemisphere (Ebersbach et al., 2017; Pan et al., 2001; Tkach et al., 2015). Recent molecular phylogenetic research recognized at least 13 sections and nine subsections within the genus (Tkach et al., 2015). *Saxifraga* sect. *Irregulares* Haw., characterized by asymmetric flowers with two unequally elongated and three short petals, was one of the earliest lineage of *Saxifraga* to diverge (Magota et al., 2021; Soltis et al., 2001; Tkach et al., 2015; Zhang et al., 2020). It is distributed in eastern Asia, and many species are narrow endemics only known from a few localities. (Magota et al., 2021; Pan et al., 2001). Section *Irregulares* currently comprises 20 species, including seven recently described from China (14 species in China currently). (Wang et al., 2008; Zhang et al., 2017, 2018, 2019, 2021, 2022; Zhao et al., 2019). The diversity of *S*. sect. *Irregulares* was previously underestimated as only seven species of *S*. sect. *Irregulares* were recorded in *Flora of China* (Pan et al., 2001), and more investigations and phylogenetic analyses are needed to clarify the patterns of variation and the delimitation of species (Zhang et al., 2021). The purpose of this study was to reevaluate the taxonomic and phylogenetic status of taxa that were associated with or synonymized under *S. mengtzeana* Engl. & Irmsch. in the account of this species in *Flora Reipublicae Popularis Sinicae* (Pan, 1992) and in *Flora of China* (Pan et al., 2001).

**FIGURE 1:**
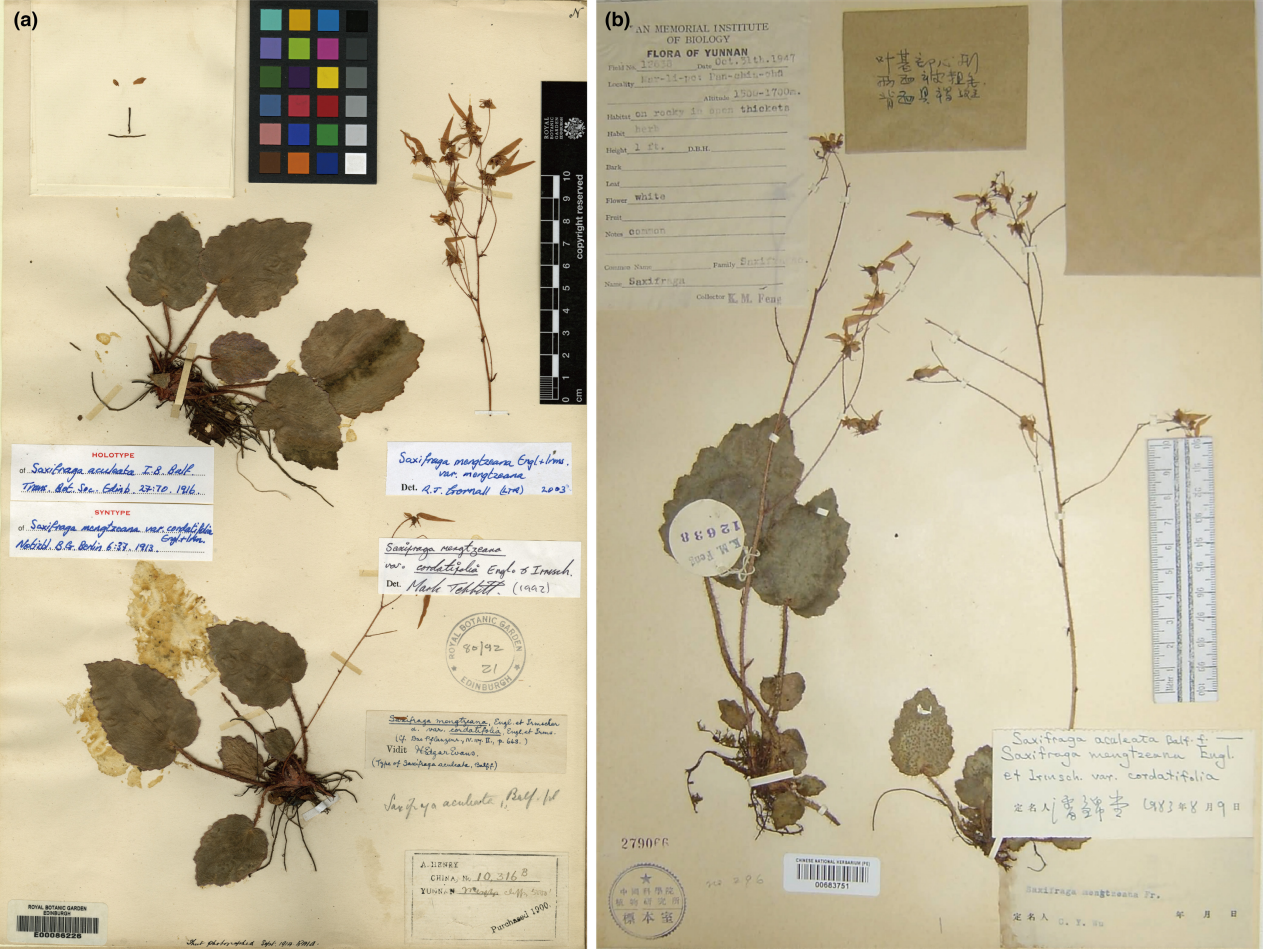
(a) Lectotype of *Saxifraga mengtzeana* Engl. & Irmsch. (*A. Henry 10316B* [E]) and (b) isotype of *S. epiphylla* Gornall & H.Ohba (*K.M. Feng12638* [PE]).

Taxa of particular focus in this study are *S. mengtzeana*, *S. epiphylla* Gornall & H.Ohba and *S. geifolia* Balf.f. *Saxifraga mengtzeana* var. *cordatifolia* Engl. & Irmsch. (1913) and *Saxifraga aculeata* Balf.f. (1916) have the same type (*A. Henry 10316B*, Figure 1a), while *S. mengtzeana* var. *peltifolia* Engl. & Irmscher (1913) and *Saxifraga henryi* Balf.f. (1916) were described based on the same collection (*A. Henry 9118*), only *S. mengtzeana* needs to be considered of these on species level (Balfour, 1916; Engler & Irmscher, 1913; Gornall et al., 2000; Pan et al., 2001; Taxonomic treatment section).

Variants with a foliar embryo in the basal leaf sinus were not mentioned by Engler & Irmscher (1913, 1916/19) nor by Balfour (1916), but they seem to have been first recognized by Pan (1992), who misapplied the name *S. aculeata* to them. The type collections of *S. aculeata*, however, lack foliar embryos and, as explained earlier, are the same taxon as the type of *S. mengtzeana* (Gornall et al., 2000). The viviparous taxon was distinguished as a separate species and given its first formal name as *S. epiphylla* Gornall & H. Ohba (Gornall et al., 2000) based on the collection by *Feng 12638* (Figure 1b). Soon afterwards, Chuang (2001) described *S. mengtzeana* var. *foliolata* H. Chuang for the taxon with foliar embryos; Chuang cited *Wang 82433* as the holotype but included *Feng 12638* among the paratypes, thus ensuring synonymy of the two names.

**FIGURE 2:**
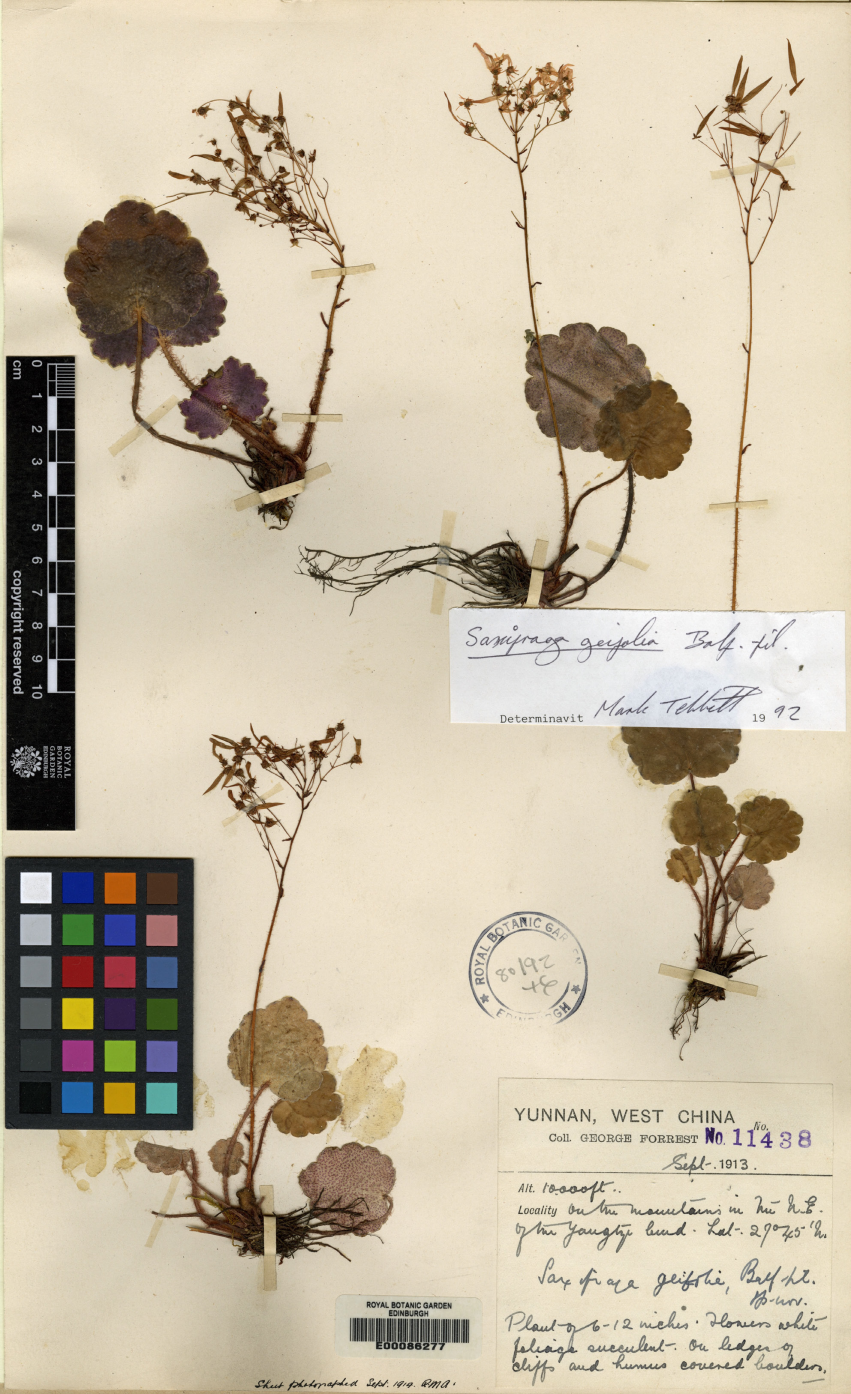
Lectotype of *Saxifraga geifolia* Balf.f. (*G.Forrest 11438* [E]).

Type material of *S. mengtzeana* and *S. epiphylla* comes from southeastern Yunnan. The only two synonymized taxa from outside southern Yunnan are *S. geifolia* Balf.f. and *Saxifraga ovatocordata* Hand.‐Mazz. (the latter from western Sichuan and not considered further here). Balfour (1916) described *S. geifolia* based on *G. Forrest 11438* (Figure 2) from lat. 27°45′N in northwestern Yunnan. It was included in *S. mengtzeana* in the *Flora of China* (Pan et al., 2001), although morphologically, the leaf shape of the holotype specimen is rounder than the triangular‐cordate to ovate leaf shape of *S. mengtzeana*. Geographically, the type locality of *S. geifolia* is some 500 km distant from the type locality of *S. mengtzeana*. The morphological and geographical differences suggest that *S. geifolia* may be distinct rather than conspecific with *S. mengtzeana*.

## 2 MATERIALS AND METHODS

### 2.1 Phylogenetic reconstruction

We sampled 22 collections representing *Saxifraga mengtzeana* and related taxa, of which 20 were newly sequenced and two were obtained from GenBank (Table 1). Leaf materials were collected in the field and from dried herbarium specimens, voucher information and GenBank accession numbers were presented in Table 1. *Saxifraga sinomontana* from *Saxifraga* sect. *Ciliatae* was selected as the outgroup, based on previously proposed phylogenetic relationships based on molecular analyses (Tkach et al., 2015).

**TABLE 1:** Voucher information and GenBank accessions for phylogenetic analysis.

Taxon	Locality	Voucher	GenBank accession number	
			*Plastid genome*	*ITS*
*Saxifraga geifolia* 1	SC: Garzê	deng10896 (KUN)	OQ428204	OQ428929
*Saxifraga geifolia* 2	NY: Lijiang	deng11665 (KUN)	OQ428205	OQ428932
*Saxifraga geifolia* 3#	SC: Chengdu	zwy‐972 (SYS)	OQ428202	OQ428918
*Saxifraga geifolia* 4#	NY: Lijiang	deng11135 (KUN)	OQ129933	OQ428915
*Saxifraga geifolia* 5	NY: Diqing	deng12605 (KUN)	OQ428206	OQ428933
*Saxifraga mengtzeana* 1#	SY: Pingbian	zhangxj106 (KUN)	OQ406246	OQ428931
*Saxifraga mengtzeana* 2#	SY: Malipo	zhangxj110 (KUN)	OQ406247	OQ428930
*Saxifraga mengtzeana* 3	SY: Maguan	zhangxj104 (KUN)	OQ406248	OQ428934
*Saxifraga mengtzeana* 4	SY: Maguan	zhangxj177 (KUN)	OQ406249	OQ428935
*Saxifraga viridiflora* 1	Guangxi	deng12030 (KUN)	OQ428208	OQ428926
*Saxifraga viridiflora* 2	Guangxi	zhangxj98 (KUN)	OQ428207	OQ428925
*Saxifraga damingshanensis*	Guangxi	zwy‐1208 (SYS)	OQ428203	OQ428924
*Saxifraga daqiaoensis*	Guangdong	deng12102 (KUN)	OQ405944	OQ428919
*Saxifraga kegangii*	Hunan	BJ4668 (JIU)	OQ405946	OQ428921
*Saxifraga kwangsiensis*	Guangxi	deng12168 (KUN)	OQ405945	OQ428920
*Saxifraga luoxiaoensis*	Hunan	LXP‐13‐16785(SYS)	OQ405947	OQ428922
*Saxifraga shennongii*	Hunan	LXP‐09‐09089	OQ434240	OQ428923
*Saxifraga rufescens*	NY: Diqing	deng13173 (KUN)	OQ129932	OQ428917
*Saxifraga spp*. guangxi1	Guangxi	deng11976 (KUN)	OQ428209	OQ428927
*Saxifraga spp*. guangxi2	Guangxi	deng12140 (KUN)	OQ428210	OQ428928
*Saxifraga sinomontana*		/	MN104589	MH432876
*Saxifraga stolonifera*		/	MN496079	OQ428916

Abbreviations: #, individuals with foliar embryos; NY, Northern Yunnan; SC, Sichuan; SY, Southeastern Yunnan.

Total genomic DNA was extracted from leaf material using DP305 Plant Genomic DNA kits (Tiangen, Beijing, China) following the manufacturer's protocol. Sequencing libraries were generated using the NEB Next® Ultra DNA Library Prep Kit for Illumina® (NEB, USA). The prepared libraries were sequenced on an Illumina Hiseq 4000 platform with 150 bp paired‐end reads. Plastid genome data and nrDNA sequences were assembled using GetOrganelle pipeline (Jin et al., 2020). Complete plastomes were annotated in batches using PGA v.3 (Qu et al., 2019). A concatenation‐based approach was conducted for the plastid coding sequences for 73 shared protein coding genes of 22 samples, and sequences were aligned in MAFFT 7 (Katoh et al., 2019). Partial nrDNA sequences include internal transcribed spacers ITS1 and ITS2 of nuclear ribosomal DNA and the 5.8 S rRNA gene (ITS region). Phylogenetic reconstruction was performed using maximum likelihood (ML) and Bayesian inference (BI). Maximum likelihood analysis was implemented in IQ‐Tree with 1000 bootstrap (BS) replicates to assess clade support (Nguyen et al., 2015). As identified by jModeltest 2.1.7, the GTR + I + G and SYM + G model of sequence evolution was selected using the Akaike information criterion (AIC) for BI analyses of plastid dataset and ITS sequences, respectively (Posada, 2008). Bayesian analysis used MrBayes version 3.2.6 (Huelsenbeck & Ronquist, 2001). Four parallel Markov Chains Monte Carlo (MCMC) simulations were run and sampled every 1000 generations for 20 million generations in total, with the first 25% trees discarded as burn‐in, and runs were considered to have converged to stationarity when their average standard deviation of split frequencies was <0.01. (Ronquist et al., 2012).

### 2.2 Morphological comparison

**FIGURE 3:**
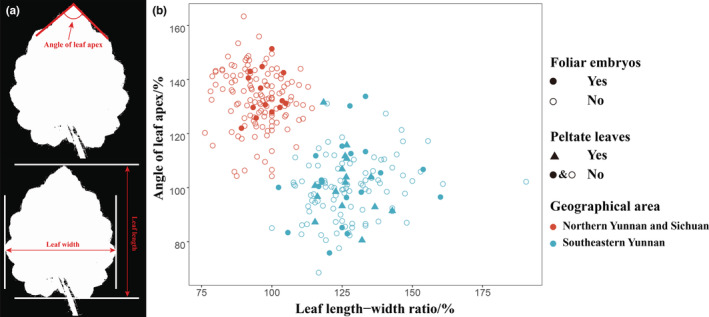
(a) Measuring the angle of leaf apex and leaf length relative to width; (b) Comparison of leaf characters for georeferenced *Saxifraga mengtzeana* (type locality and other sampling sites within southeastern Yunnan) and *S. geifolia* (type locality and other sampling sites from northern Yunnan and Sichuan), (Drawn by Quan‐Sheng Fu).

Morphological data were recorded from field collections, cultivations, and herbarium specimens. Voucher specimens of our collections were deposited in the herbarium of the Kunming Institute of Botany (KUN), Kunming, China. Herbarium specimens of *Saxifraga mengtzeana* and related taxa were examined in CDBI, CSFI, GXMG, IBSC, KUN, PE, and SM (acronyms follow Thiers, 2018), either by examining the specimens directly or by examining their digital images provided by the National Plant Specimen Resource Center (http://www.cvh.ac.cn/index.php), and JSTOR Global Plants web portal (https://plants.jstor.org/). Leaf length relative to width (aspect ratio) and the angle of leaf apex (Figure 3) were measured for quantitative analysis (Appendix S1). In addition, observations were made of the base of leaf blade (cordate or peltate, viviparous or not), the shape of the leaf apex (subacute vs. obtuse), the leaf texture (thick, leathery and slightly succulent vs. papery or slightly leathery), and markings on the adaxial leaf surface (concolorous vs. with white or virescent streaks). Voucher specimens for the morphological observations are cited under Additional specimens examined in the taxonomic treatment and in Table 1.

## 3 RESULTS

### 3.1 Phylogenetic analyses

**FIGURE 4:**
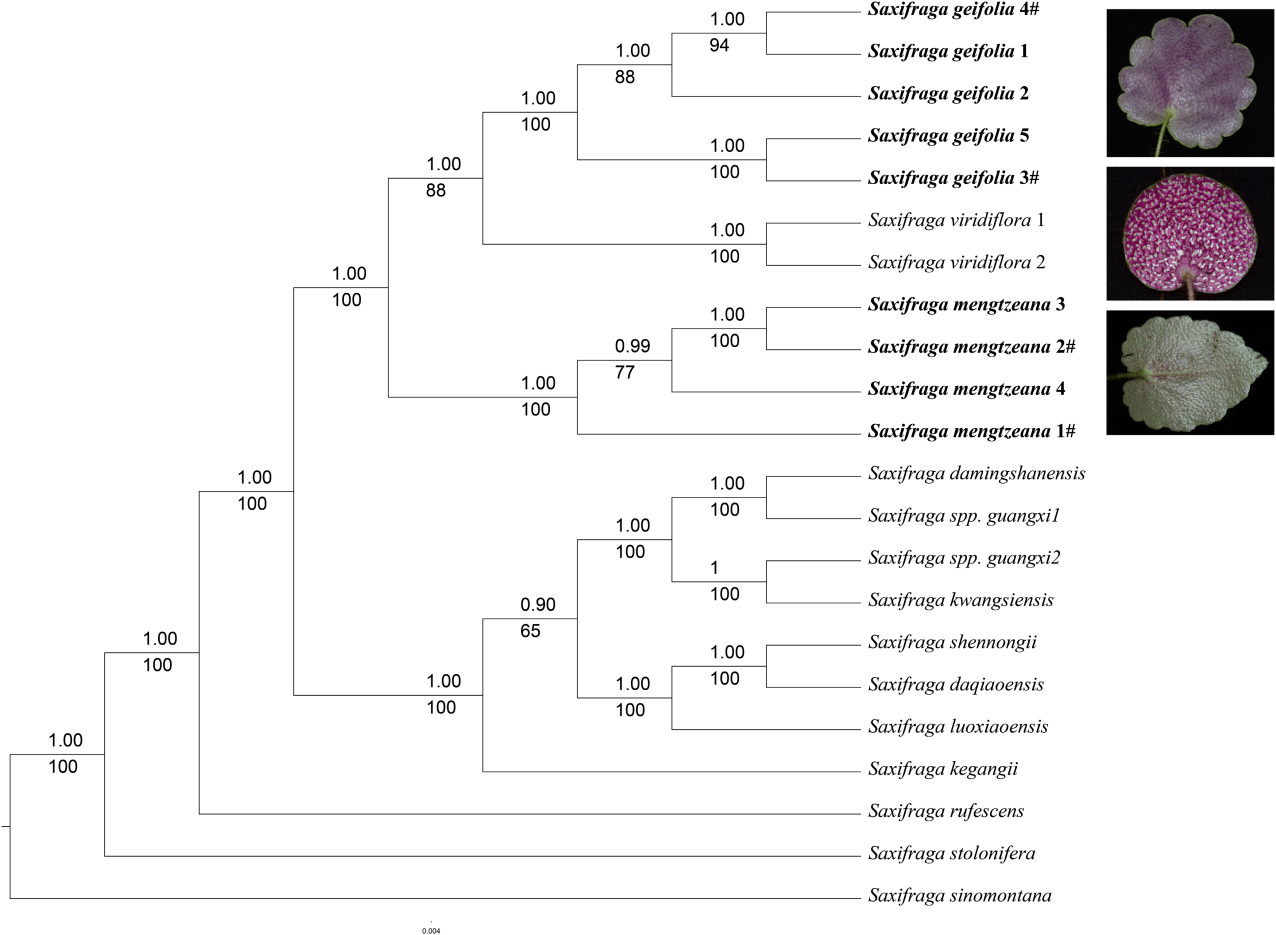
Bayesian consensus tree of species of *Saxifraga* sect. *Irregulares* derived from the combined 73 plastid coding regions dataset., with *S. sinomontana* as outgroup. Numbers above branches indicate ML bootstraps, numbers below branches are Bayesian posterior probability. **#:** individuals with foliar embryos.

Fourteen taxa were included in the phylogenetic analysis using 73 shared protein coding plastid genes (CDS) of 22 samples (Figure 4). The resulting concatenated matrix dataset contained 60,399 bp. The 50% majority‐rule consensus tree based on maximum likelihood bootstraps (MLBS) and Bayesian posterior probability (BIPP) of the plastid DNA sequences both showed that five accessions from northwestern Yunnan and Sichuan (*Saxifraga geifolia*), two individuals with foliar embryos and three without foliar embryos, formed a monophyletic clade with a strong support (BIPP = 1.00, MLBS = 100) and were sister to *Saxifraga viridiflora* (BIPP = 1.00, MLBS = 88). Four accessions from southeastern Yunnan, two individuals with foliar embryos (previously *Saxifraga epiphylla*) and two without foliar embryos (*S. mengtzeana*), grouped together (BIPP = 1.00, MLBS = 100) and were sister to the clade of *S. geifolia* + *S. viridiflora* (BIPP = 1.00, MLBS = 100). Specimens previously recorded as *S. mengtzeana* (*S*. spp. guangxi1 and *S*. spp. guangxi2) from Guilin of Guangxi Province grouped together with *Saxifraga damingshanensis* + *Saxifraga kwangsiensis* and were sister to the clade of *S. shennongii* + *S. daqiaoensis* + *S. luoxiaoensis*, but the support for this group is weak (BIPP = 0.90, MLBS = 65).

The resulting multiple alignment of the ITS region was 660 bp. The 50% majority‐rule consensus tree based on Bayesian posterior probability (BIPP) and maximum likelihood bootstraps (MLBS) of the ITS sequences both showed that five accessions of *S. geifolia* grouped together (BIPP = 1, MLBS = 100), sister to *S. mengtzeana* with strong supports (BIPP = 0.99, MLBS = 94). There was hard incongruence at deeper nodes between plastid and ITS phylogenies (Figure 4, Figure S1). The ITS phylogeny indicated a sister relationship between *S. geifolia* and *S. mengtzeana*, and was sister to the clade of *S. daqiaoensis* + *S. shennongii* + *S. luoxiaoensis* + *S. kegangii* + *S. damingshanensis* + *S. kwangsiensis* + *S*. sp. guangxi1 + *S*. sp. guangxi2, but with low support rates (BIPP = 0.69, MLBS = 66). Besides, in the ITS phylogeny, *Saxifraga viridiflora* was sister to the clade of all species mentioned above, rather than sister to *S. geifolia* in plastid phylogeny (Figure S1).

### 3.2 Morphology

Analysis of the morphological variation shows that it can be divided into two reasonably well‐marked groups (Figure 3) based on leaf outline: (a) a northern group with rotund to ovate, more or less isodiametric leaves with bluntly crenate margins, and (b) a southeastern group with triangular‐ovate leaves, longer than wide, with more coarsely dentate margins. Plants with a peltate petiole insertion are restricted to the southeastern group, but those with foliar embryos occur in both groups (Figure 3).

**FIGURE 5:**
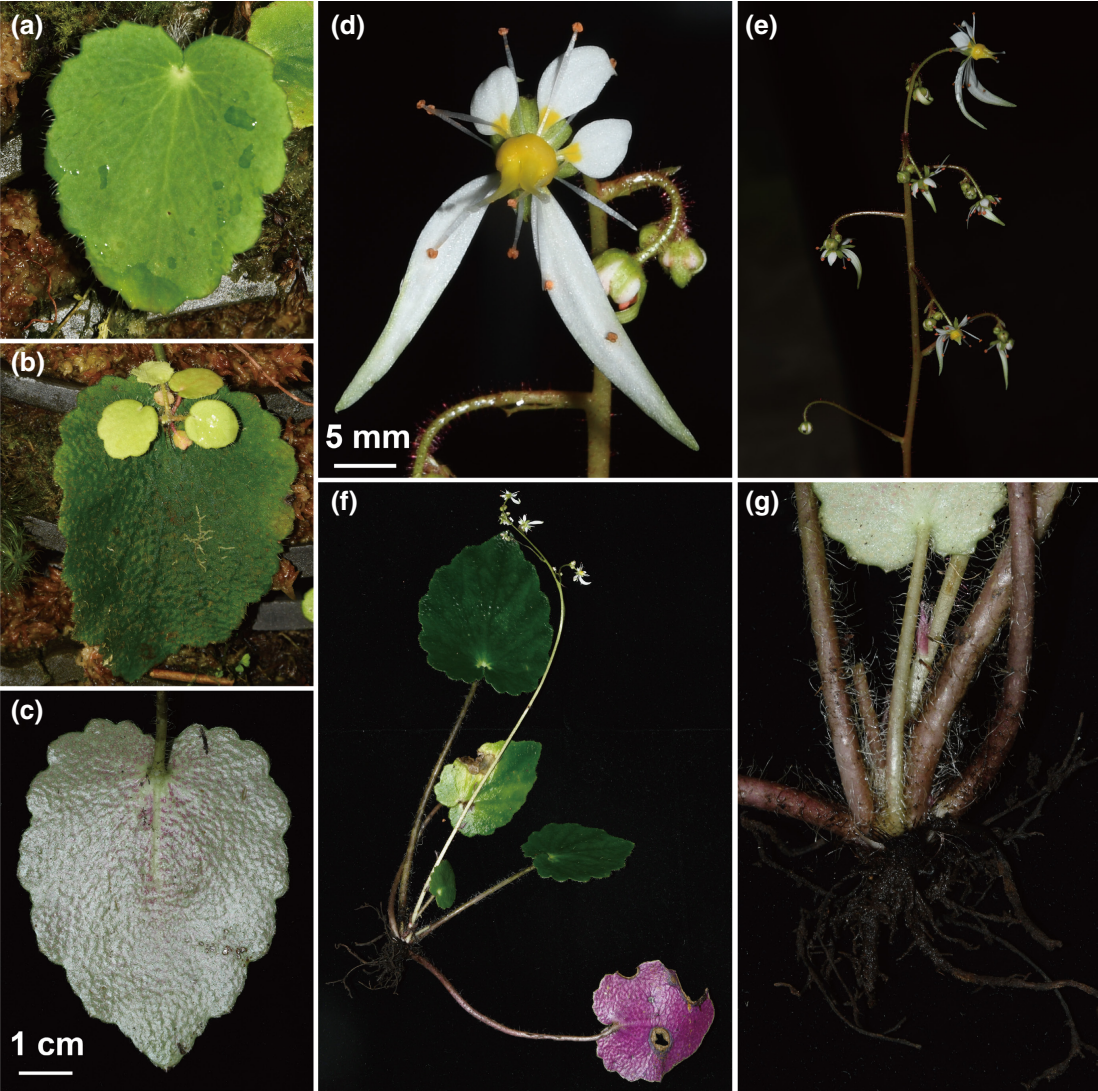
*Saxifraga mengtzeana* from the type locality of *S. epiphylla* in Malipo. (a) Leaf blade without foliar embryo; (b) Leaf blade with foliar embryo; (c) Abaxial leaf surface; (d) Flower; (e) Inflorescence; (f) Plant; (g) Rhizomes.

**FIGURE 6:**
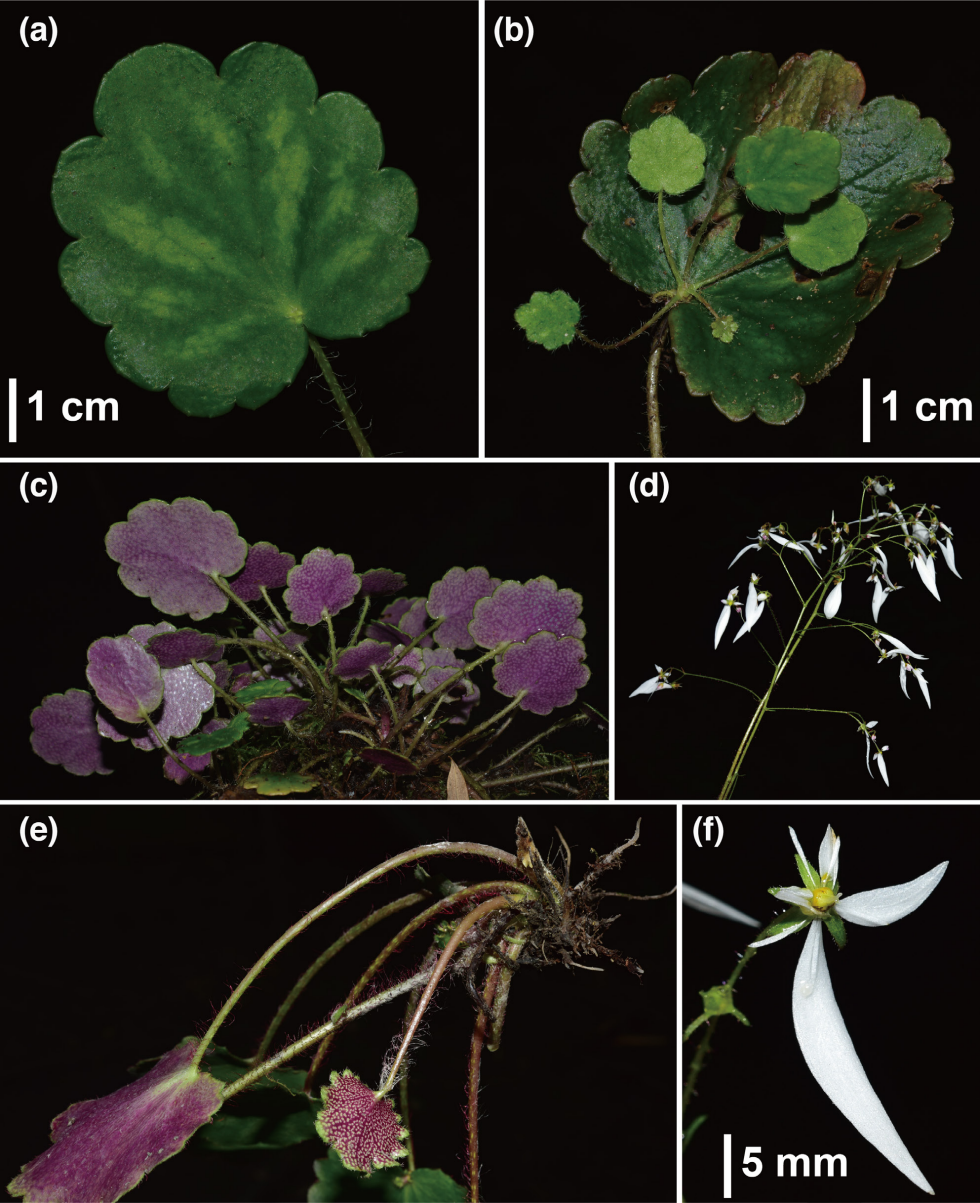
*Saxifraga geifolia* from type locality. (a) Leaf blade without foliar embryo, with virescent streaks; (b) Leaf blade with foliar embryo; (c) Abaxial leaf surface; (d) Inflorescence; (e) Rhizomes; (f) Flower.

In addition to the differences in leaf shape, other distinctive features associated with the geographical groups include the leaf apex (subacute in the south vs. obtuse in the north), leaf texture (thick, leathery, and slightly succulent in the south vs. papery or slightly leathery in the north), and markings on the adaxial leaf surface (concolorous in the south vs. with white or virescent streaks in the north) (Figures 5 and 6). Comparison with type specimens shows that the southern group corresponds to *Saxifraga mengtzeana* (including *S. epiphylla*) and the northern group corresponds to *S. geifolia*. The southeastern populations flower from September to November, whereas those in the north flower May to September.

**FIGURE 7:**
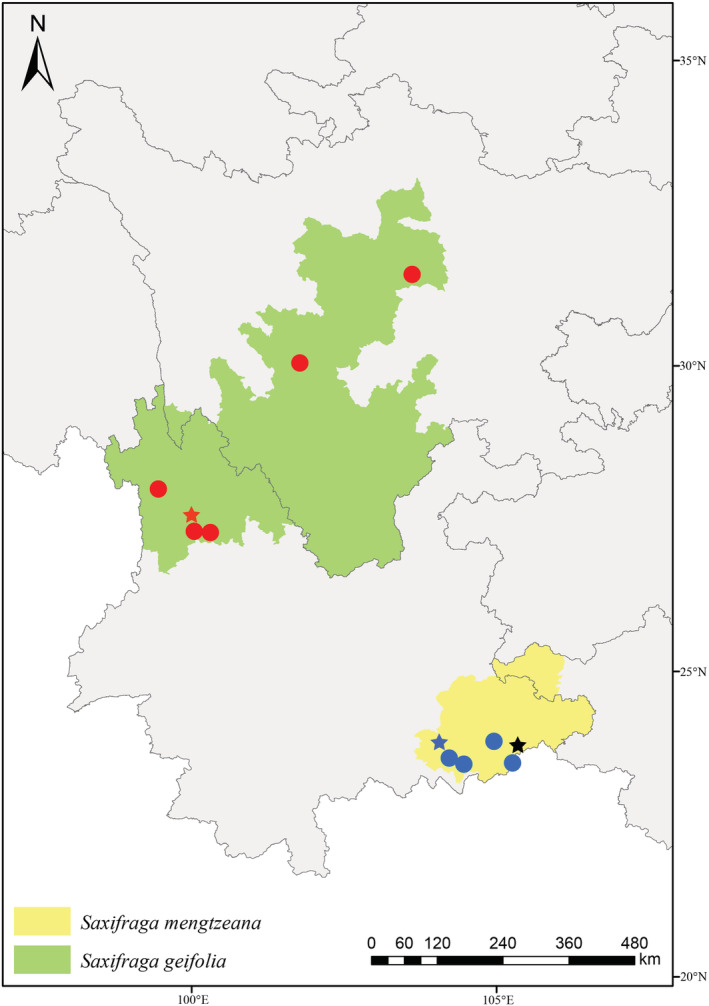
Distribution of *Saxifraga mengtzeana* (yellow region) and *S. geifolia* (green region) according to field collections and herbarium specimens, with sampling sites of *S. mengtzeana* (blue dots) and *S. geifolia* (red dots) for plants used in phylogenetic analyses. Type localities of *S. geifolia* (red star), *S. mengtzeana* (blue star), *S. epiphylla* (black star) indicated. (Drawn by Xin‐Yuan Kuai).

Observations on field collections, cultivations, and herbarium specimens found that plants with a peltate petiole insertion are restricted to the southeastern group and co‐occur with phenotypes that have petioles inserted at the base of the leaf‐blades, while plants with foliar embryos occur in populations of both *S. mengtzeana* and *S. geifolia*, alongside plants without such embryos (Figure 3, Appendix S1). Field studies at the type locality of *S. epiphylla* in Malipo County, Yunnan, showed that, even here, the population is polymorphic for this feature. Sometimes, the embryos are very small, reduced to a tiny, leafless bud. Furthermore, we found foliar embryos in some plants in PingBian County, which is adjacent to Mengtze, the type locality of *S. mengtzeana* (Figure 7). Examination of specimens of these two species, including the types, shows very little difference apart from the foliar embryos (Figure 1, Table 2).

**TABLE 2:** Comparison of *Saxifraga mengtzeana*, *S. epiphylla*, *S. geifolia*, and *S. viridiflora* from morphological data.

Characters	*Saxifraga mengtzeana*	*Saxifraga epiphylla*	*Saxifraga geifolia*	*Saxifraga viridiflora*
Stolons	Absent	Absent	Absent	Absent
Foliar embryo	Present or not	Present or not	Present or not	Absent
Leaf blade	Triangular‐cordate to ovate; base cordate or peltate; apex subacute	Triangular‐cordate; base cordate; apex subacute	Reniform; base cordate; apex obtuse	Reniform; base cordate; apex obtuse
Leaf texture	Slightly succulent	Thick leathery	Papery/slightly leathery	Thick leathery
Leaf margin	Coarsely dentate	Coarsely dentate	Crenate‐lobed	Undulate
Flowering time	September to November	September to November	May to September	April to July
Distribution	Southeastern Yunnan	Southeastern Yunnan	Northern Yunnan, Sichuan	Northern Guangxi

## 4 DISCUSSION

Morphological and molecular patterns of variation are consistent in distinguishing a northern taxon from a southeastern taxon. The northern taxon corresponds to *Saxifraga geifolia*, with rotund to ovate, more or less isodiametric, cordate leaves with bluntly crenate margins, an obtuse leaf apex, a papery or only slightly leathery texture, and whose adaxial surface is streaked with white or pale green. The southeastern taxon matches *S. mengtzeana*, with triangular‐ovate (longer than wide), cordate leaves, with more coarsely dentate margins and a subacute apex, a thick, leathery and slightly succulent texture, and a concolorous adaxial surface. Despite some morphological overlap between the northern and southeastern populations, the differences are sufficient to warrant the recognition of two distinct species, particularly in light of molecular support. Although significant cytonuclear discordances were inferred, our plastid and ITS datasets both indicated a separation between the northern taxon (*S. geifolia*) and southeastern taxon (*S. mengtzeana*). The two genepools are likely reproductively isolated from one another not only by geographical distance but also by flowering time, with the northern populations blooming in summer, from May to September, and the southeastern ones in the autumn, September to November. Additionally, phylogenetic incongruence between plant nuclear and plastid genomes may provide evidence of introgression (Folk et al., 2017), but more evidences are needed to investigate the introgression and/or hybridization among *Irregulares* species. Besides, specimens previously recorded as *S. mengtzeana* (*S*. spp. guangxi1 and *S*. spp. guangxi2) from Guilin of Guangxi Province were found to be closer to *S. kwangsiensis* and *S. damingshanensis* in both plastid and ITS trees with low support rates and topological incongruence. Hybridization and/or introgression may occur in these four taxa, as all of them were endemic to Guangxi Province. *Saxifraga* spp. guangxi1 and *S*. spp. guangxi2 were usually identified as *S. mengtzeana* despite obvious difference in leaf shape. It may lie in the fact that only seven species of *Irregulares* were recorded in *Flora of China* (Pan et al., 2001), and only *S. mengtzeana* was characterized by stolons absent, leaf blade ovate and spotted abaxially, leaf without foliar embryos. However, several new species of *Irregulares* were reported in recent years, and in many of them, stolons and embryos were absent, leaf blade ovate and spotted abaxially (Wang et al., 2008; Zhang et al., 2017, 2018, 2019, 2021; Zhao et al., 2019).

The difference of the base of leaf blade (cordate or peltate) in *S. mengtzeana* is not clear‐cut, because the point of insertion of the petiole can vary such that intermediate phenotypes occur. Furthermore, observations of plants of the related, peltate‐leaved *Saxifraga daqiaoensis* in the field and cultivation, show that the peltate feature is developmentally plastic with new leaves having basifixed petioles and only in later leaves are the petioles in a peltate position; and individuals may vary from 1 year to the next. The degree to which the petioles are peltate of *S. mengtzeana* var. *peltifolia* is variable, and there are intermediates with the basifixed phenotypes; there is a possibility that the character is developmentally labile. We propose, therefore, to abandon it as a taxon and synonymize it fully under *S. mengtzeana*.

The case of *S. epiphylla* is somewhat different. The trait of a leaf blade with foliar embryos in the adaxial sinuses is stable within a plant from year to year and is presumably therefore genetically determined. Its pattern of occurrence, however, whereby it occurs apparently somewhat randomly within populations of *S. mengtzeana*, suggests that the foliar embryos are not diagnostic of a species. Indeed, Zhang et al. (2020) compared the morphology of herbarium specimens of *S. mengtzeana* and *S. epiphylla* from KUN and PE and found no features to support their separation except the presence of foliar embryos. Molecular data also strongly suggest that plants with foliar embryos do not form a monophyletic group (Figure 4). Since the character appears to be under genetic control and is an important feature of the phenotype, scattered within populations, we propose that taxonomic recognition is warranted at the rank of forma. This is easily accomplished in the case of *S. mengtzeana* where a valid description and a type specimen exists, but a new taxon needs to be established for the viviparous plants of *S. geifolia*.

## 5 TAXONOMIC TREATMENT

***Saxifraga mengtzeana*** Engl. et Irmsch., Notizbl. Königl. Bot. Gart. Berlin. 6(51): 36. 1913.

**Type**: China, Yunnan, Mengtzi, auf Felsen 1500–1800 m. *A. Henry 10316* (syntype B, presumed destroyed; isosyntypes E!, MO); *A. Henry 10316B* (lectotype E! (Gornall et al., 2000); isolectotypes MO!, PE!; syntype B, presumed destroyed).

**Homotypic synonyms**: *Saxifraga mengtzeana* var. *cordatifolia* Engl. & Irmsch., Notizbl. Königl. Bot. Gart. Berlin. 6(51): 37. 1913.

*Saxifraga aculeata* Balf. f., Trans. Bot. Soc. Edinburgh. 27(1): 70. 1916. Lectotype E! (*A. Henry 10316B*: (Gornall et al., 2000); isolectotypes MO!, PE!; syntypes B, presumed destroyed). Gornall et al. (2000) referred to the Edinburgh specimen of this collection as the holotype, since that is where I.B. Balfour worked. However, since the species was published before 1990 and duplicate types exist in other herbaria, the attribution of type status must be formalized by means of a lectotypification, provided here; see McNeill (2014).

*Diptera mengtzeana* (Engl. & Irmsch.) Losinsk, Bull. Jard. Bot. Princ. URSS 27: 601. 1928.

**Heterotypic synonyms**: *Saxifraga mengtzeana* var. *peltifolia* Engl. & Irmsch., Notizbl. Königl. Bot. Gart. Berlin. 6(51): 37. 1913. TYPE: China, Yunnan, Mengtze, um 1800 m. *A. Henry 9118* (holotype B, presumed destroyed; lectotype MO!, designated here; iso(lecto)types E!, NY, US).

*Saxifraga henryi* Balf. f., Trans. Bot. Soc. Edinburgh. 27(1): 72. 1916. TYPE: China, Yunnan, Mengtz, southwest mountains. *A. Henry 9118*. (lectotype E!, designated here, or perhaps holotype; iso(lecto)types MO!, NY, US).

**Description:** Herbs, perennial, 10–40 cm tall. Stolons absent. Rhizomes short. Leaves all basal; petiole 5–15 cm long, brown glandular hairy, barely sheathing at base; leaf blade triangular‐cordate to ovate, thick leathery and slightly succulent, 4.5–8.5 cm long × 3.5–7.0 cm wide, both surfaces sparsely hispid or nearly glabrous, abaxially greenish or purple, with brown or purple spots, base cordate or peltate, occasionally with a foliar embryo in sinus, margin coarsely dentate, apex subacute. Inflorescence paniculate, ca. 25 cm long. 10–30‐flowered; branches 2.4–5.0 cm long, glandular pubescent, 2–4‐flowered; pedicels slender, 1.0–2.0 cm long, glandular pubescent. Flowers zygomorphic; sepals 5, spreading to reflexed, oblong‐ovate, 2.0–3.0 mm long × 1.0–1.5 mm wide, adaxially glabrous, abaxially and marginally glandular pubescent. Petals 5, white, margin entire; shortest 3 petals triangular‐ovate, 2.5–4.0 mm long × 1.8–2.2 mm wide, apex acute to shortly acuminate; longer petal narrowly ovate, 8.0–12.0 mm long × 1.5–2.7 mm wide; longest petal sublanceolate, 15.0–25.0 mm long × 3.0–5.5 mm wide. Stamens 10, 3.5–4.5 mm long. Ovary ovoid, 1.5–2.0 mm long, with a semiannular nectary disc; styles divergent ca. 1.5–1.8 mm long. Seeds elliptic, surfaces slightly bent, ca. 0.5 mm long.

**Distribution**: China (Yunnan: Honghe, Wenshan; Guangxi: Longlin).

**Additional specimens examined**: **China. Yunnan:** Yanshan County, 15 November 1939, *C.W. Wang 84961* (KUN); Yanshan County, 18 November 1939, *C.W. Wang 85101* (KUN); Malipo County, 31 October 1947, *K.M. Feng 12638* (KUN); Maguan County, 8 October 2021, *X.J. Zhang zhangxj104* (KUN); **Guangxi:** Longlin County, 28 October 2014, *H.Z. Lv, D.X. Nong & H.F. Cen 451031141028053LY* (GXMG).

***Saxifraga mengtzeana*** f. ***epiphylla*** (Gornall & H.Ohba) X‐J. Zhang & Gornall, comb. et stat. nov.

**Basionym**: *Saxifraga epiphylla* Gornall & H. Ohba, Novon. 10(4): 375. 2000. TYPE: China. Yunnan. Malipo. *K‐M. Feng 12638* (holotype A!; isotypes KUN!, PE!, WUK).

**Synonym**: *Saxifraga mengtzeana* var. *foliolata* H. Chuang, Acta Bot. Yunnan. 23(2): 159. 2001. TYPE: China, Yunnan, Pingbian, *C‐W. Wang 82433* (holotype KUN1205296!; isotypes KUN1205298!, PE!); Pingbian County, 28 September 1939, *C‐W. Wang 82165* (paratypes KUN!, PE!); Yunnan, Malipo, *K‐M. Feng 12638* (paratypes A!, KUN!, PE!, WUK).

**Diagnosis**: *Saxifraga mengtzeana* f. *epiphylla* differs from *S. mengtzeana* f. *mengtzeana* in having foliar embryos in the sinus of the basal leaves.

**Distribution**: China (Yunnan: Honghe, Wenshan).

**Additional specimens examined**: **China. Yunnan:** Mengtze City, 11 October 1939, C.W. Wang 83396 (KUN); Pingbian County, 8 October 2021, *X.J. Zhang zhangxj106* (KUN); Malipo County, 4 December 2021, *X.J. Zhang zhangxj110* (KUN).

***Saxifraga geifolia* Balf.f.**, Trans. Bot. Soc. Edinburgh. 27(1): 72. 1916.

**Type:** China, Yunnan, on ledges of cliffs and humus‐covered boulders; on the mountains in the north‐east of the Yangtze bend. Lat. 27°45′N, September 1913, *G. Forrest 11438*. (lectotype E!, designated here; isolectotypes KUN!, PE!).

**Description:** Herbs, perennial, 15–36 cm tall. Stolons absent. Rhizomes short. Leaves all basal; petiole 5–12 cm long, brown glandular hairy, barely sheathing at the base; leaf blade reniform, papery or slightly leathery, 3–6.5 cm long × 3.0–5.0 cm wide, both surfaces glandular hispid or nearly glabrous, abaxially greenish or purple, with brown spots, base cordate, occasionally with a foliar embryo in sinus, margin crenate‐lobed, lobes crenulate or subentire, apex obtuse. Inflorescence paniculate, ca. 30 cm long. 15–30‐flowered; branches 3.0–7.0 cm long, glandular pubescent, 3–5‐flowered; pedicels slender, 1.0–2.0 cm long, glandular pubescent. Flowers zygomorphic; sepals 5, spreading, oblong, 2.0–3.5 mm long × 1.5–2.0 mm wide, adaxially glabrous, abaxially and marginally glandular pubescent. Petals 5, white, margin entire; shortest 3 petals ovate, 2.0–4.0 mm long × 1.5–2.5 mm wide, apex acute to shortly acuminate; longer petal lanceolate, 8.0–15.0 mm long × 1.8–3.0 mm wide; longest petal linear‐lanceolate to lanceolate, 12.0–20.0 mm long × 2.2–5.5 mm wide. Stamens 10, 3.5–4.2 mm long. Ovary ovoid, 1.5–2.0 mm long, with a semiannular nectary disc; styles divergent ca. 1.3–1.8 mm long. Seeds elliptic, surfaces slightly bent, ca. 0.5 mm long.

**Distribution:** China (northern Yunnan, Sichuan).

**Additional specimens examined: China. Sichuan:** Tianquan County, 14 May 1980, *Q.Q. Wang 21876* (IBSC); Dechang County, 2 July 1976, *Northwest Normal University Biology Department No. 12007* (PE); Xide County, 24 May 1979, *No. 0335* (SM); Ganluo County, 17 June 1979, *No. 514* (SM); Ganluo County, 23 September 1976, *No. 14325* (PE); Meigu County, 12 June 1979, *No. 242* (SM); Jinyang County, 7 August 1978, *No. 466* (SM); Huili County, 2 July 1968, *Group H. Yun No. 56* (SM); Dujiangyan City, 26 May 2014, *J.J. Zhou 140526002* (CSFI); Kangding City, 27 June 1965, *Y.T. Zhang & K.Y. Lang 32* (PE); Kangding City, 8 August 1959, *S. Jiang & C.L. Jin* 02739 (PE); Baoxing County, 12 May 1959, *X.S. Zhang & Y.X. Ren 4595* (PE); Tianquan County, 18 April 1953, *X.L. Jiang 33880* (PE); Luding County, 3 July 1934, *C.S. Liu 632* (PE); Leibo County, 9 May 1979, *Leibo Exped. 156* (SM); Barkam City, 22 July 1960, *No. 22299* (SM); Heishui County, 8 June 1959, No. 1325 (SM); Xingwen County, 12 May 1959, No. 0341 (KUN); Danba County, 20 July 1959, No. 02212 (KUN); Mountain Omei, 25 November 1940, *W.P. Fang 15545* (KUN); Yuexi County, 28 June 1959, *No. 3509* (KUN); Wenchuan County, 30 May 2021, *J.T. Chen, J.Y. Peng & X.J. Zhang deng11135* (KUN); Jinchuan County, 27 May 2021, *J.T. Chen, J.Y. Peng & X.J. Zhang deng10990* (KUN); **Yunnan:** Western Yunnan, 1930, *G. Forrest 28748* (PE); Shangri‐La, 11 June 1981, *QTP Exped. 795* (KUN); Shangri‐La, 6 November 1939, *K.M. Feng 3247* (KUN); Gongshan County, 29 May 1960, *No. 8795* (KUN); Gongshan County, 1 September 1940, *K.M. Feng 7277* (KUN); Lijiang City, 19 June 2021, *T. Deng, J.T. Chen, J.Y. Peng & X.J. Zhang deng11665* (KUN); Gongshan County, 20 July 2021, *H. Sun, T. Deng, X.H. Huang deng12605* (KUN); Dêqên County, 22 September 2021, *H. Sun, T. Deng, J.T. Chen & Q. Liu deng13387* (KUN); Weixi County, 20 May 2022, *X.J. Zhang zhangxj160* (KUN).

***Saxifraga geifolia*** f. ***vivipara*** X‐J. Zhang & Gornall, f. nov.

**Type:** China. Yunnan. Lijiang City, Naxi Autonomous County of Yulong. 27°28′N, 99°25′E, 2844 m alt., 29 August 2020, *P.J. Liu, J.T. Chen, Q. Liu & X.J. Zhang deng10084* (holotype KUN!).

**Diagnosis**: *Saxifraga geifolia* f. *vivipara* differs from *S. geifolia* f. *geifolia* in having foliar embryos in the sinus of the basal leaves.

**Distribution:** China (Northern Yunnan, Sichuan).

**Additional specimens examined: China. Yunnan:** Lijiang City, 20 June 2021, *T. Deng, J.T. Chen, J.Y. Peng & X.J. Zhang deng11787* (KUN); Sichuan: Chengdu City, Dujiangyan, Mount Qingcheng, 09 June 2021, *J.T. Chen, J.Y. Peng & X.J. Zhang deng11441* (KUN).

## AUTHOR CONTRIBUTIONS

**Xin‐Jian Zhang:** Conceptualization (lead); formal analysis (lead); writing – original draft (lead); writing – review and editing (equal). **Richard J. Gornall:** Formal analysis (equal); writing – review and editing (equal). **Zhuo‐Xin Zhang:** Formal analysis (equal); writing – review and editing (equal). **Jun‐Tong Chen:** Investigation (supporting); writing – review and editing (supporting). **Hang Sun:** Funding acquisition (lead); project administration (lead); supervision (lead). **Tao Deng:** Funding acquisition (lead); supervision (supporting); writing – review and editing (supporting).

## CONFLICT OF INTEREST STATEMENT

The authors declare that there is no conflict of interest.

## Supporting information


Figure S1.

Appendix S1.

## Data Availability

The sequences of this study have been deposited in The National Center for Biotechnology Information (NCBI) database. GenBank accession numbers of the sequencing data can be found in Table 1. The data used for quantitative analysis of morphological comparison are included in Appendix S1.
