# Supplementary figures and images for: A re‐examination of the circumscription of Saxifraga mengtzeana (Saxifragaceae)
Source: Ecol Evol. 2023 Mar 12;13(3):e9886. doi: 10.1002/ece3.9886 (PMC10008273; doi:10.1002/ece3.9886)

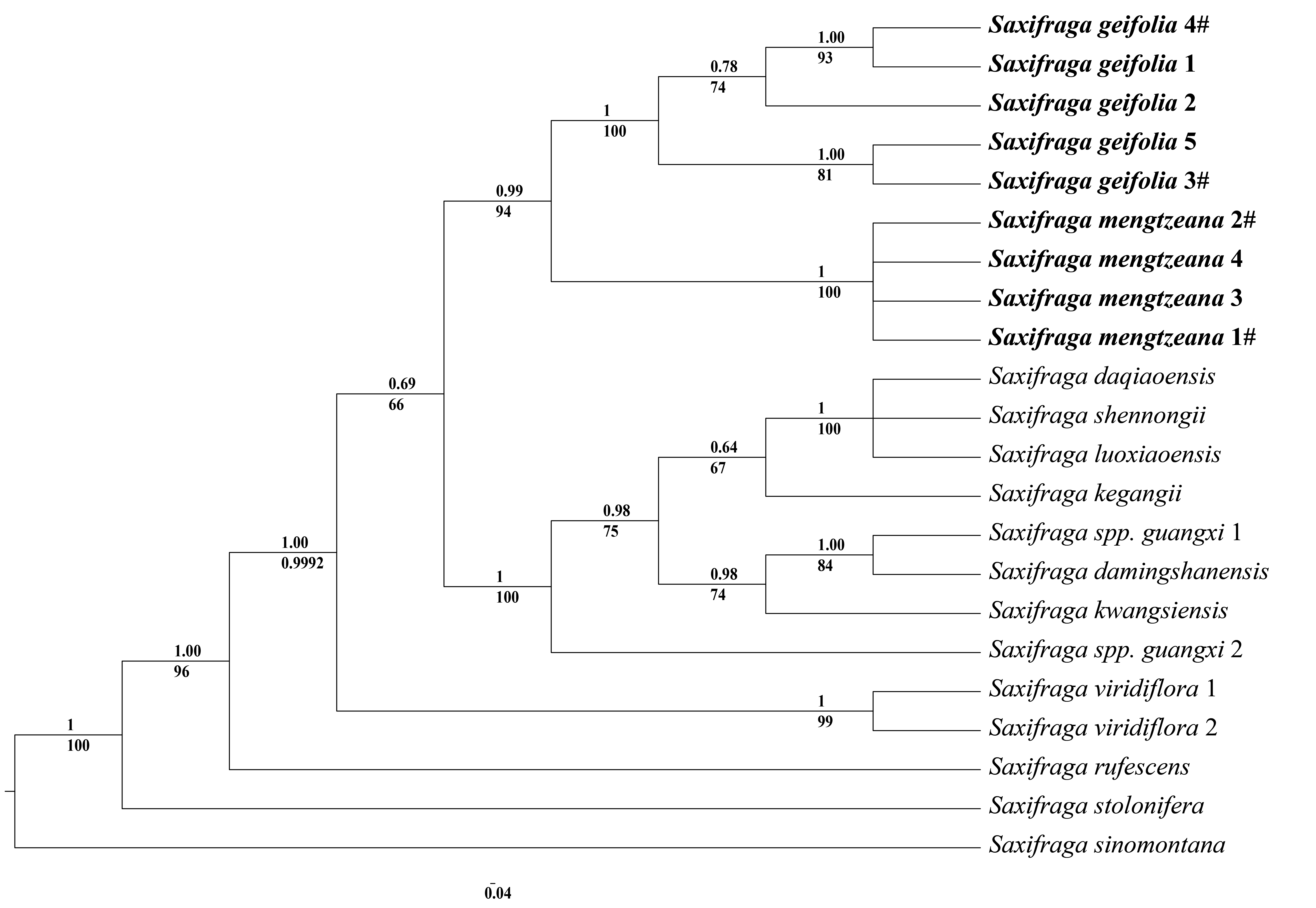

Supplement: Supplementary file 1 — Figure S1. [file ECE3-13-e9886-s002.png]
