# Supplementary material for: A re‐examination of the circumscription of Saxifraga mengtzeana (Saxifragaceae)
Source: Ecol Evol. 2023 Mar 12;13(3):e9886. doi: 10.1002/ece3.9886 (PMC10008273; doi:10.1002/ece3.9886)
Supplement: Supplementary file 2 — Appendix S1. [file ECE3-13-e9886-s001.docx]

**Appendix A** Sampling data for quantitative analysis of morphological comparison

| Geographical area | Voucher | leaf length-width ratio | angle of leaf apex | Foliar embryos | Peltate leaves |
| --- | --- | --- | --- | --- | --- |
| SY | A Henry 9118 (NY) | 126.47 | 103.87 | × | √ |
| SY | A Henry 9118 (NY) | 136.67 | 92.85 | × | √ |
| SY | A Henry 9118 (NY) | 142.86 | 91.40 | × | √ |
| SY | A Henry 9118 (NY) | 135.29 | 103.90 | × | √ |
| SY | A Henry 9118 (US) | 126.09 | 111.70 | × | √ |
| SY | A Henry 9118 (US) | 122.73 | 98.51 | × | √ |
| SY | A Henry 9118 (US) | 115.38 | 100.73 | × | √ |
| SY | A Henry 9118 (US) | 126.19 | 101.89 | × | √ |
| SY | A Henry 9118 (US) | 118.42 | 131.55 | × | √ |
| SY | A Henry 10316 (E) | 127.59 | 98.77 | × | × |
| SY | A Henry 10316 (E) | 125.00 | 118.64 | × | × |
| SY | A Henry 10316 (E) | 117.89 | 102.48 | × | × |
| SY | A Henry 9118 (E) | 125.00 | 93.24 | × | √ |
| SY | A Henry 9118 (E) | 115.38 | 87.23 | × | √ |
| SY | A Henry 9118 (E) | 117.54 | 82.46 | × | × |
| SY | A Henry 9118 (E) | 132.08 | 80.54 | × | √ |
| SY | A Henry 9118 (E) | 116.22 | 96.67 | × | √ |
| SY | A Henry 10316 (PE) | 105.71 | 83.38 | √ | × |
| SY | A Henry 10316 (PE) | 126.56 | 96.34 | √ | × |
| SY | A Henry 10316 (PE) | 128.57 | 85.21 | × | × |
| SY | A Henry 10316 (PE) | 125.00 | 99.23 | × | × |
| SY | A Henry 10316B (PE) | 118.18 | 93.28 | × | × |
| SY | A Henry 10316B (PE) | 126.47 | 99.83 | × | × |
| SY | A Henry 10316B (PE) | 122.00 | 95.53 | × | × |
| SY | A Henry 10316B (PE) | 116.33 | 95.10 | × | × |
| SY | A Henry 10316B (PE) | 133.33 | 99.43 | × | × |
| SY | A Henry 10316B (PE) | 113.33 | 91.18 | × | × |
| SY | A Henry 10316B (PE) | 137.93 | 87.49 | × | × |
| SY | A Henry 10316B (PE) | 140.91 | 98.50 | × | × |
| SY | A Henry 10316B (PE) | 127.66 | 82.32 | × | × |
| SY | A Henry 10316B (E) | 125.81 | 97.96 | × | × |
| SY | A Henry 10316B (E) | 124.44 | 90.00 | × | × |
| SY | A Henry 10316B (E) | 123.91 | 77.59 | × | × |
| SY | A Henry 10316B (E) | 137.84 | 102.77 | × | × |
| SY | A Henry 10316B (E) | 112.50 | 86.46 | × | × |
| SY | A Henry 10316B (E) | 128.13 | 92.94 | × | × |
| SY | A Henry 10316B (E) | 126.83 | 101.14 | × | × |
| SY | A Henry 10316B (E) | 123.53 | 107.02 | × | × |
| SY | C.W.Wang 83396 (KUN) | 125.00 | 115.37 | √ | × |
| SY | C.W.Wang 83396 (KUN) | 114.89 | 97.62 | × | × |
| SY | C.W.Wang 83396 (KUN) | 127.78 | 108.95 | × | × |
| SY | C.W.Wang 83396 (KUN) | 150.00 | 102.46 | × | × |
| SY | C.W.Wang 83396 (KUN) | 133.33 | 102.73 | × | × |
| SY | C.W. Wang 82165 (KUN) | 126.83 | 82.95 | √ | × |
| SY | C.W. Wang 82165 (KUN) | 142.86 | 92.38 | × | × |
| SY | C.W. Wang 82165 (KUN) | 132.35 | 100.87 | × | × |
| SY | C.W. Wang 82165 (KUN) | 120.00 | 116.93 | × | × |
| SY | C.W. Wang 82165 (KUN) | 121.88 | 92.25 | × | × |
| SY | C.W. Wang 82165 (KUN) | 125.00 | 85.23 | √ | × |
| SY | C.W. Wang 82165 (KUN) | 133.33 | 113.34 | √ | × |
| SY | C.W. Wang 82165 (KUN) | 127.78 | 130.25 | √ | × |
| SY | C.W. Wang 82165 (KUN) | 147.37 | 97.82 | × | × |
| SY | C.W. Wang 82165 (KUN) | 116.67 | 100.46 | √ | × |
| SY | zhangxj106 (KUN) | 142.86 | 91.29 | × | × |
| SY | zhangxj106 (KUN) | 121.21 | 100.33 | × | × |
| SY | zhangxj106 (KUN) | 125.00 | 106.86 | × | √ |
| SY | zhangxj106 (KUN) | 133.33 | 86.58 | × | × |
| SY | zhangxj106 (KUN) | 117.65 | 102.80 | √ | × |
| SY | zhangxj106 (KUN) | 134.48 | 98.74 | × | × |
| SY | zhangxj106 (KUN) | 146.67 | 104.53 | × | × |
| SY | zhangxj106 (KUN) | 138.89 | 110.34 | × | × |
| SY | zhangxj106 (KUN) | 117.86 | 102.04 | √ | × |
| SY | zhangxj106 (KUN) | 118.92 | 101.67 | × | × |
| SY | zhangxj106 (KUN) | 126.67 | 110.75 | × | √ |
| SY | zhangxj106 (KUN) | 113.16 | 108.21 | × | × |
| SY | zhangxj106 (KUN) | 123.08 | 83.09 | × | × |
| SY | zhangxj106 (KUN) | 122.22 | 99.95 | × | × |
| SY | zhangxj106 (KUN) | 116.13 | 101.35 | × | × |
| SY | zhangxj106 (KUN) | 128.57 | 86.69 | × | × |
| SY | K.M. Feng12638 (PE) | 120.51 | 75.84 | √ | × |
| SY | K.M. Feng12638 (PE) | 107.84 | 101.10 | × | × |
| SY | K.M. Feng12638 (PE) | 100.00 | 85.04 | × | × |
| SY | K.M. Feng12638 (WUK) | 102.38 | 100.09 | √ | × |
| SY | K.M. Feng12638 (WUK) | 132.26 | 91.81 | × | × |
| SY | K.M. Feng12638 (WUK) | 125.00 | 87.68 | × | × |
| SY | K.M. Feng12638 (KUN) | 133.33 | 105.41 | × | × |
| SY | K.M. Feng12638 (KUN) | 146.67 | 117.14 | × | × |
| SY | K.M. Feng12638 (KUN) | 160.87 | 101.35 | × | × |
| SY | K.M. Feng12638 (KUN) | 155.56 | 117.26 | × | × |
| SY | K.M. Feng12638 (KUN) | 190.48 | 102.18 | × | × |
| SY | zhangxj110 (KUN) | 136.36 | 111.25 | × | × |
| SY | zhangxj110 (KUN) | 133.33 | 85.91 | × | × |
| SY | zhangxj110 (KUN) | 112.90 | 93.11 | × | × |
| SY | zhangxj110 (KUN) | 146.15 | 121.36 | × | × |
| SY | zhangxj110 (KUN) | 140.54 | 127.41 | × | × |
| SY | zhangxj110 (KUN) | 144.44 | 112.28 | × | × |
| SY | zhangxj110 (KUN) | 160.00 | 96.52 | √ | × |
| SY | zhangxj110 (KUN) | 152.94 | 105.90 | × | × |
| SY | zhangxj110 (KUN) | 126.67 | 115.71 | × | √ |
| SY | zhangxj110 (KUN) | 142.86 | 107.40 | × | × |
| SY | zhangxj110 (KUN) | 119.23 | 80.56 | × | × |
| SY | zhangxj110 (KUN) | 122.73 | 87.31 | × | × |
| SY | zhangxj110 (KUN) | 125.00 | 108.92 | × | × |
| SY | zhangxj110 (KUN) | 138.71 | 105.49 | √ | × |
| SY | zhangxj110 (KUN) | 129.17 | 110.39 | × | × |
| SY | zhangxj110 (KUN) | 108.33 | 111.20 | × | × |
| SY | zhangxj110 (KUN) | 133.33 | 133.78 | √ | × |
| SY | zhangxj110 (KUN) | 115.63 | 111.80 | √ | × |
| SY | zhangxj110 (KUN) | 119.15 | 100.30 | × | × |
| SY | zhangxj110 (KUN) | 153.85 | 106.74 | √ | × |
| SY | zhangxj110 (KUN) | 118.75 | 102.53 | × | × |
| SY | K.M. Feng 12638 (KUN) | 116.67 | 68.55 | × | × |
| SY | K.M. Feng 12638 (KUN) | 108.33 | 100.72 | × | × |
| SY | K.M. Feng 12638 (KUN) | 140.00 | 110.52 | × | × |
| SY | C.W. Wang 84961 (KUN) | 122.73 | 97.13 | × | × |
| SY | C.W. Wang 84961 (KUN) | 148.00 | 87.02 | × | × |
| SY | C.W. Wang 84961 (KUN) | 116.67 | 114.57 | × | × |
| SY | C.W. Wang 84961 (KUN) | 116.39 | 108.16 | × | × |
| SY | C.W. Wang 84961 (KUN) | 110.71 | 86.13 | × | × |
| SY | C.W. Wang 84961 (KUN) | 119.23 | 112.27 | × | × |
| SY | C.W. Wang 84961 (KUN) | 131.82 | 98.33 | √ | × |
| SY | C.W. Wang 84961 (KUN) | 119.64 | 131.10 | × | × |
| SY | C.W. Wang 84961 (KUN) | 110.53 | 82.75 | × | × |
| SY | C.W. Wang 84961 (KUN) | 113.89 | 97.24 | × | × |
| SY | C.W. Wang 84961 (KUN) | 138.46 | 101.66 | × | × |
| SY | C.W. Wang 84961 (KUN) | 128.13 | 112.62 | √ | × |
| SY | C.W. Wang 84961 (KUN) | 131.71 | 101.46 | × | × |
| SY | C.W. Wang 84961 (KUN) | 135.48 | 103.59 | × | × |
| SY | C.W. Wang 85101 (KUN) | 122.22 | 111.99 | × | × |
| SY | C.W. Wang 85101 (KUN) | 124.39 | 102.61 | × | × |
| SY | C.W. Wang 85101 (KUN) | 107.69 | 101.92 | × | × |
| SY | C.W. Wang 85101 (KUN) | 136.00 | 106.18 | × | × |
| SY | C.W. Wang 85101 (KUN) | 126.09 | 85.56 | × | × |
| SY | C.W. Wang 85101 (KUN) | 114.29 | 95.20 | × | × |
| SY | C.W. Wang 85101 (KUN) | 127.27 | 83.23 | × | × |
| SY | C.W. Wang 85101 (KUN) | 125.42 | 99.38 | × | × |
| SY | C.W. Wang 85101 (KUN) | 115.38 | 94.51 | × | × |
| SY | C.W. Wang 85101 (KUN) | 110.71 | 102.34 | × | × |
| NYS | deng10084 (KUN) | 93.75 | 126.02 | × | × |
| NYS | deng10084 (KUN) | 86.11 | 135.49 | × | × |
| NYS | deng10084 (KUN) | 91.67 | 140.60 | √ | × |
| NYS | deng10084 (KUN) | 103.45 | 141.90 | × | × |
| NYS | deng10084 (KUN) | 98.55 | 142.10 | × | × |
| NYS | deng10084 (KUN) | 92.31 | 126.70 | × | × |
| NYS | deng10084 (KUN) | 97.67 | 130.68 | √ | × |
| NYS | deng10084 (KUN) | 98.08 | 138.01 | × | × |
| NYS | deng10084 (KUN) | 100.00 | 133.24 | × | × |
| NYS | deng10084 (KUN) | 102.86 | 129.69 | √ | × |
| NYS | deng10084 (KUN) | 78.18 | 135.64 | × | × |
| NYS | deng10084 (KUN) | 91.18 | 130.40 | × | × |
| NYS | deng10084 (KUN) | 109.68 | 138.00 | × | × |
| NYS | deng10084 (KUN) | 105.00 | 131.19 | √ | × |
| NYS | deng10084 (KUN) | 93.33 | 129.69 | √ | × |
| NYS | deng10084 (KUN) | 104.55 | 130.19 | × | × |
| NYS | deng10084 (KUN) | 87.50 | 130.78 | × | × |
| NYS | deng10084 (KUN) | 92.31 | 132.38 | × | × |
| NYS | deng10084 (KUN) | 95.45 | 127.80 | × | × |
| NYS | deng10084 (KUN) | 104.17 | 142.56 | √ | × |
| NYS | deng10084 (KUN) | 95.65 | 131.42 | × | × |
| NYS | deng10084 (KUN) | 94.74 | 111.46 | × | × |
| NYS | deng10084 (KUN) | 96.00 | 136.84 | √ | × |
| NYS | deng10084 (KUN) | 85.71 | 138.95 | × | × |
| NYS | deng10084 (KUN) | 97.44 | 139.37 | × | × |
| NYS | deng10084 (KUN) | 100.00 | 135.03 | × | × |
| NYS | deng10084 (KUN) | 90.63 | 147.17 | × | × |
| NYS | deng10084 (KUN) | 84.38 | 148.53 | × | × |
| NYS | deng10084 (KUN) | 89.19 | 122.01 | √ | × |
| NYS | deng10084 (KUN) | 100.00 | 132.65 | × | × |
| NYS | deng10084 (KUN) | 86.21 | 141.42 | × | × |
| NYS | Deng11787 (KUN) | 96.67 | 122.48 | × | × |
| NYS | Deng11787 (KUN) | 103.70 | 132.14 | √ | × |
| NYS | Deng11787 (KUN) | 97.44 | 145.89 | × | × |
| NYS | Deng11787 (KUN) | 92.31 | 143.03 | √ | × |
| NYS | Deng11787 (KUN) | 96.55 | 144.79 | √ | × |
| NYS | Deng11787 (KUN) | 85.71 | 136.10 | × | × |
| NYS | Deng11787 (KUN) | 104.65 | 126.33 | × | × |
| NYS | Deng11787 (KUN) | 100.00 | 127.48 | × | × |
| NYS | Deng11787 (KUN) | 92.68 | 136.88 | × | × |
| NYS | Deng11787 (KUN) | 100.00 | 128.00 | √ | × |
| NYS | Deng11787 (KUN) | 100.00 | 137.55 | × | × |
| NYS | Deng11787 (KUN) | 90.48 | 143.89 | × | × |
| NYS | Deng11787 (KUN) | 92.31 | 139.79 | × | × |
| NYS | G.Forrest 11438 (E) | 90.32 | 106.34 | × | × |
| NYS | G.Forrest 11438 (E) | 105.56 | 138.45 | × | × |
| NYS | G.Forrest 11438 (E) | 112.90 | 137.32 | × | × |
| NYS | G.Forrest 11438 (E) | 100.00 | 143.86 | × | × |
| NYS | G.Forrest 11438 (E) | 106.90 | 133.62 | × | × |
| NYS | G.Forrest 11438 (E) | 91.94 | 147.13 | × | × |
| NYS | G.Forrest 11438 (E) | 107.14 | 139.13 | × | × |
| NYS | G.Forrest 11438 (E) | 108.70 | 115.02 | × | × |
| NYS | G.Forrest 11438 (E) | 100.00 | 151.39 | √ | × |
| NYS | G.Forrest 11438 (E) | 94.44 | 140.19 | × | × |
| NYS | G.Forrest 11438 (E) | 102.70 | 134.86 | × | × |
| NYS | G.Forrest 11438 (E) | 98.25 | 119.29 | × | × |
| NYS | G.Forrest 11438 (E) | 93.55 | 124.71 | × | × |
| NYS | G.Forrest 11438 (PE) | 93.94 | 118.81 | × | × |
| NYS | G.Forrest 11438 (PE) | 100.00 | 126.62 | × | × |
| NYS | G.Forrest 11438 (PE) | 85.71 | 145.45 | × | × |
| NYS | G.Forrest 11438 (PE) | 91.53 | 142.92 | × | × |
| NYS | G.Forrest 11438 (PE) | 88.00 | 118.99 | × | × |
| NYS | G.Forrest 11438 (PE) | 98.53 | 121.02 | × | × |
| NYS | G.Forrest 11438 (KUN) | 100.00 | 104.23 | × | × |
| NYS | G.Forrest 11438 (KUN) | 97.56 | 109.56 | × | × |
| NYS | G.Forrest 11438 (KUN) | 101.64 | 141.34 | × | × |
| NYS | G.Forrest 11438 (KUN) | 87.50 | 104.30 | × | × |
| NYS | deng11441 (KUN) | 83.78 | 141.34 | × | × |
| NYS | deng11441 (KUN) | 94.44 | 122.64 | × | × |
| NYS | deng11441 (KUN) | 100.00 | 106.60 | × | × |
| NYS | deng11441 (KUN) | 109.68 | 125.03 | × | × |
| NYS | deng11441 (KUN) | 100.00 | 122.70 | × | × |
| NYS | deng11441 (KUN) | 90.24 | 125.72 | × | × |
| NYS | deng11441 (KUN) | 106.67 | 132.31 | × | × |
| NYS | deng11441 (KUN) | 88.00 | 131.12 | × | × |
| NYS | deng11441 (KUN) | 108.70 | 131.02 | × | × |
| NYS | deng11441 (KUN) | 103.23 | 140.41 | × | × |
| NYS | deng11441 (KUN) | 94.44 | 134.18 | × | × |
| NYS | deng11441 (KUN) | 94.44 | 125.78 | √ | × |
| NYS | deng11441 (KUN) | 97.30 | 126.33 | × | × |
| NYS | deng11441 (KUN) | 85.71 | 120.56 | × | × |
| NYS | deng11441 (KUN) | 78.57 | 132.51 | × | × |
| NYS | deng11441 (KUN) | 91.43 | 145.36 | × | × |
| NYS | ZZL 87-0961 (PE) | 100.00 | 121.21 | × | × |
| NYS | ZZL 87-0961 (PE) | 100.00 | 113.53 | × | × |
| NYS | ZZL 87-0961 (PE) | 105.56 | 121.56 | × | × |
| NYS | ZZL 87-0961 (PE) | 78.95 | 115.56 | × | × |
| NYS | ZZL 87-0961 (PE) | 89.29 | 118.11 | × | × |
| NYS | ZZL 87-0961 (PE) | 76.19 | 120.29 | × | × |
| NYS | ZZL 87-0961 (PE) | 95.45 | 155.96 | × | × |
| NYS | ZZL 87-0961 (PE) | 105.00 | 126.87 | × | × |
| NYS | ZZL 87-0961 (PE) | 98.48 | 137.73 | × | × |
| NYS | ZZL 87-0961 (PE) | 110.23 | 144.00 | × | × |
| NYS | ZZL 87-0961 (PE) | 100.00 | 124.51 | × | × |
| NYS | ZJJ 140526002 (CSFI) | 104.35 | 137.40 | × | × |
| NYS | ZJJ 140526002 (CSFI) | 92.31 | 137.73 | × | × |
| NYS | ZJJ 140526002 (CSFI) | 96.00 | 134.51 | × | × |
| NYS | ZJJ 140526002 (CSFI) | 94.12 | 123.69 | × | × |
| NYS | ZJJ 140526002 (CSFI) | 111.11 | 125.52 | × | × |
| NYS | ZJJ 140526002 (CSFI) | 93.94 | 143.48 | × | × |
| NYS | ZJJ 140526002 (CSFI) | 103.70 | 135.47 | × | × |
| NYS | ZJJ 140526002 (CSFI) | 98.11 | 130.17 | × | × |
| NYS | Y&W 206 (CDBI) | 96.15 | 128.43 | × | × |
| NYS | Y&W 206 (CDBI) | 114.29 | 129.40 | × | × |
| NYS | Y&W 206 (CDBI) | 97.56 | 127.79 | × | × |
| NYS | Y&W 206 (CDBI) | 100.00 | 149.55 | × | × |
| NYS | Zhangxu637 (KUN) | 80.00 | 142.87 | × | × |
| NYS | Zhangxu637 (KUN) | 78.79 | 141.21 | × | × |
| NYS | Zhangxu637 (KUN) | 101.75 | 124.07 | × | × |
| NYS | Zhangxu637 (KUN) | 96.43 | 133.89 | × | × |
| NYS | Zhangxu637 (KUN) | 90.00 | 135.76 | × | × |
| NYS | Zhangxu637 (KUN) | 90.00 | 163.40 | × | × |
| NYS | Zhangxu637 (KUN) | 80.00 | 143.24 | × | × |
| NYS | Zhangxu637 (KUN) | 82.35 | 143.39 | × | × |
| NYS | Zhangxu771 (KUN) | 88.00 | 153.43 | × | × |
| NYS | Zhangxu771 (KUN) | 92.86 | 136.33 | × | × |
| NYS | Zhangxu771 (KUN) | 82.50 | 136.47 | × | × |
| NYS | Z&L 32 (PE) | 82.35 | 143.77 | × | × |
| NYS | Z&L 32 (PE) | 91.67 | 148.72 | × | × |
| NYS | Z&L 32 (PE) | 86.67 | 145.01 | × | × |
| NYS | Z&L 32 (PE) | 91.07 | 147.99 | × | × |
| NYS | Jiang&Jin 02739 (PE) | 85.45 | 128.33 | × | × |
| NYS | Jiang&Jin 02739 (PE) | 86.96 | 139.62 | × | × |
| NYS | Jiang&Jin 02739 (PE) | 88.89 | 141.62 | × | × |
| NYS | QTP 795 (KUN) | 98.08 | 147.77 | × | × |
| NYS | QTP 795 (KUN) | 100.00 | 133.09 | × | × |
| NYS | QTP 795 (KUN) | 92.31 | 142.23 | × | × |
| NYS | QTP 795 (KUN) | 80.00 | 138.13 | × | × |
| NYS | QTP 795 (KUN) | 86.67 | 105.90 | × | × |
| NYS | QTP 795 (KUN) | 96.00 | 130.87 | × | × |
| NYS | QTP 795 (KUN) | 101.60 | 129.22 | × | × |
| NYS | QTP 795 (KUN) | 93.98 | 131.63 | × | × |
| NYS | QTP 795 (KUN) | 111.11 | 130.60 | × | × |
| NYS | Feng 3247 (KUN) | 90.32 | 122.83 | × | × |
| NYS | Feng 3247 (KUN) | 98.11 | 132.69 | × | × |
| NYS | Feng 3247 (KUN) | 98.89 | 133.03 | × | × |
| NYS | Feng 3247 (KUN) | 96.77 | 132.08 | × | × |
| NYS | Feng 3247 (KUN) | 101.82 | 144.15 | × | × |
| NYS | Feng 3247 (KUN) | 105.56 | 130.90 | × | × |

**Appendix B** Bayesian consensus tree of species of *Saxifraga* sect. *Irregulares* based on ITS sequences, with *S. sinomontana* as outgroup. Numbers above branches indicate ML bootstraps, numbers below branches are Bayesian posterior probability. #: individuals with foliar embryos.
